# A Novel HLA-B18 Restricted CD8+ T Cell Epitope Is Efficiently Cross-Presented by Dendritic Cells from Soluble Tumor Antigen

**DOI:** 10.1371/journal.pone.0044707

**Published:** 2012-09-06

**Authors:** Rona Y. Zhao, Nicole A. Mifsud, Kun Xiao, Kok-Fei Chan, Sara Oveissi, Heather M. Jackson, Nektaria Dimopoulos, Philippe Guillaume, Ashley J. Knights, Tamara Lowen, Neil C. Robson, Sarah E. Russell, Emmanuel Scotet, Ian D. Davis, Eugene Maraskovsky, Jonathan Cebon, Immanuel F. Luescher, Weisan Chen

**Affiliations:** 1 Ludwig Institute for Cancer Research (Melbourne Austin Branch), Austin Health, Heidelberg, Victoria, Australia; 2 Ludwig Institute for Cancer Research (Lausanne Branch), Lausanne, Switzerland; 3 Institut de Biologie, Nantes, France; 4 CSL Limited, Parkville, Victoria, Australia; Massachusetts General Hospital, United States of America

## Abstract

NY-ESO-1 has been a major target of many immunotherapy trials because it is expressed by various cancers and is highly immunogenic. In this study, we have identified a novel HLA-B*1801-restricted CD8^+^ T cell epitope, NY-ESO-1_88–96_ (LEFYLAMPF) and compared its direct- and cross-presentation to that of the reported NY-ESO-1_157–165_ epitope restricted to HLA-A*0201. Although both epitopes were readily cross-presented by DCs exposed to various forms of full-length NY-ESO-1 antigen, remarkably NY-ESO-1_88–96_ is much more efficiently cross-presented from the soluble form, than NY-ESO-1_157–165_. On the other hand, NY-ESO-1_157–165_ is efficiently presented by NY-ESO-1-expressing tumor cells and its presentation was not enhanced by IFN-γ treatment, which induced immunoproteasome as demonstrated by Western blots and functionally a decreased presentation of Melan A_26–35_; whereas NY-ESO-1_88–96_ was very inefficiently presented by the same tumor cell lines, except for one that expressed high level of immunoproteasome. It was only presented when the tumor cells were first IFN-γ treated, followed by infection with recombinant vaccinia virus encoding NY-ESO-1, which dramatically increased NY-ESO-1 expression. These data indicate that the presentation of NY-ESO-1_88–96_ is immunoproteasome dependent. Furthermore, a survey was conducted on multiple samples collected from HLA-B18^+^ melanoma patients. Surprisingly, all the detectable responses to NY-ESO-1_88–96_ from patients, including those who received NY-ESO-1 ISCOMATRIX™ vaccine were induced spontaneously. Taken together, these results imply that some epitopes can be inefficiently presented by tumor cells although the corresponding CD8^+^ T cell responses are efficiently primed *in vivo* by DCs cross-presenting these epitopes. The potential implications for cancer vaccine strategies are further discussed.

## Introduction

Professional antigen presenting cells (APC) such as dendritic cells (DCs) are responsible for the initial induction, also referred to as priming, of the cellular immune response to pathogens [1] as well as tumors [2]. Various forms of tumor antigens, soluble, cell-bound or complexed to specific antibody as immune-complex (IC), are taken up by DCs and their CD8^+^ T cell (T_CD8+_) epitopes are then presented to antigen-specific T_CD8+_ - a process called cross-presentation [3], [4], [5]. Various strategies targeting cross-presentation by DCs (such as ISCOMATRIX™ adjuvant [6]) or stimulating DC differentiation and maturation (e.g. by tumor cells expressing GM-CSF and CD40L [7]) have been developed and trialed clinically. The validity of such vaccination strategies hinges on the assumption that tumor cells display the same epitopes that are generated by the targeted DCs.

It is well established that mature DCs express the immunoproteasome constitutively [8]. However under non-immune conditions, tumor cells and other somatic cells, express the constitutive proteasome and are generally considered unable to initiate T cell responses via direct presentation due to the lack of co-stimulatory molecule expression [9]. The two types of proteasomes have been shown to cleave peptides with different specificities *in vitro* [10], [11], which is thought to lead to altered T cell selection and immune response *in vivo* to viral antigens [12], [13], [14], self antigens [11], as well as tumor antigens [15].

However, none of these studies specifically addressed cross-presentation by DCs, which is more relevant in anti-tumor immunity. It has been demonstrated in mouse models that direct antigen presentation requires continuous antigen synthesis and is typically enhanced with increased intracellular protein degradation [16], [17]; on the contrary, efficient cross-presentation relies on more stable proteins, large protein fragments [18] or ongoing protein synthesis in the antigen-donating cells [19]. It is also known that the two presentation pathways differ markedly [20]. These differences imply that DC and tumor cell present different repertoires of peptides and some of the differences may lead to disparate patterns of immune responses. For example, if given tumor antigen epitopes are not cross-presented by DCs, related immune responses may not be primed, even when tumor cells abundantly and directly present these epitopes. This scenario could provide a novel opportunity for vaccine intervention. Indeed, we have recently shown that T_CD8+_ specific for the HLA-B7-resticted NY-ESO-1_60–72_ are rarely primed under physiological conditions, yet are easily detected in melanoma patients vaccinated with NY-ESO-1 formulated with ISCOMATRIX™, a saponin and cholesterol based adjuvant that has been shown to target exogenous antigen to the cytosol to enable antigen cross-presentation [21]. Conversely, if tumor antigenic epitopes are cross-presented by DCs, but not directly presented by tumor cells, irrelevant immune responses may be primed. Such responses may not be directly protective, because the activated, tumor antigen-specific T_CD8+_ would not recognize and eliminate these tumor cells. Furthermore, the elicited T_CD8+_ could even be detrimental when they are immunodominant, because they may eliminate the cross-presenting DCs upon subsequent vaccinations and thus significantly impair priming of other subdominant T cell responses that may be beneficial to the host, a phenomenon called immunodomination [22], [23]. This scenario has potentially high clinical significance because it is difficult to alter antigen presentation by tumor cells in vivo. To date, few studies demonstrated such difference between direct- and cross-presentation for the same T_CD8+_ epitopes.

NY-ESO-1 is a cancer testis (CT) antigen expressed by a wide range of human tumors [24], [25], [26]. It is highly immunogenic both in natural disease and in vaccination settings [6], [27]. To understand the underlying mechanisms for such outstanding immunogenicity, the antigen processing and presentation properties of the NY-ESO-1 ISCOMATRIX™ vaccine and other formulations of NY-ESO-1 antigen have been characterized in our laboratories [3], [28]. In the present study, we identified and characterized another unique NY-ESO-1 epitope restricted to HLA-B*1801 (hereafter HLA-B18) from a patient who participated in our NY-ESO-1 ISCOMATRIX™ vaccine trial [6]. The novel T cell epitope, NY-ESO-1_88–96_ (LEFYLAMPF), was shown to elicit immunodominant responses and to be efficiently cross-presented by full-length, soluble NY-ESO-1 pulsed monocyte derived dendritic cells (MoDCs). However, this epitope is poorly directly presented by tumor cells expressing both HLA-B18 and NY-ESO-1, unless the immunoproteasome is expressed at high level. Our results demonstrate that not all immunodominant responses may play a direct role to eliminate tumor cells.

## Results

### Identification and Characterization of a Novel T_CD8+_ Epitope NY-ESO-1_88–96_ Presented by HLA-B18

**Figure 1:**
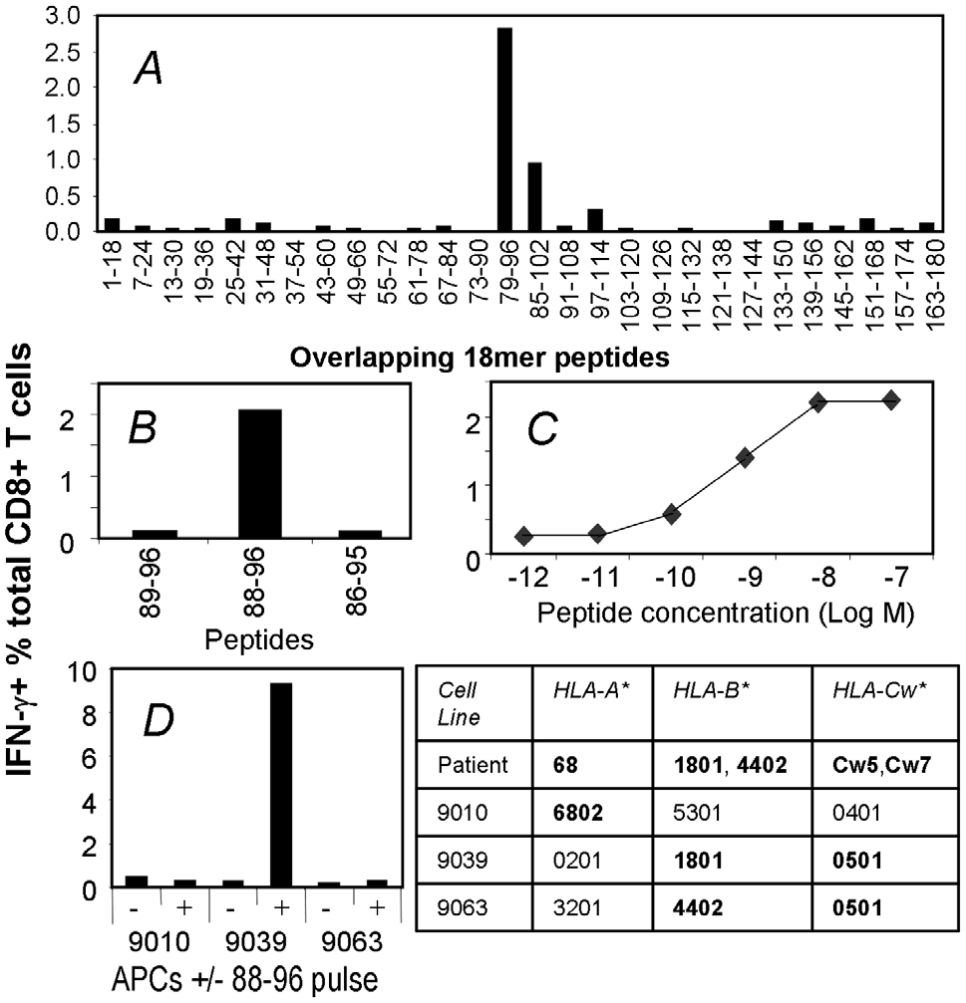
Identification and characterization of a novel NY-ESO-1 T_CD8+_ epitope. *A*. PBMCs were collected from patient 8 on day 70 following vaccination with NY-ESO-1 ISCOMATRIX™ vaccine. These cells were cultured with a panel of overlapping NY-ESO-1 18 mer peptides and then tested for responsiveness to each peptide in an ICS assay for IFN-γ. Because the background to control peptides was negligible, the results from individual cultures were plotted as a single combined figure. *B, C*, T_CD8+_ line expanded with NY-ESO-1_79–96_ 18 mer was tested under FCS-free condition for its reactivity to various HPLC-purified peptides (B) and the minimum peptide NY-ESO-1_88–96_ at various peptide concentrations (*C)*. *D*, a panel of LCL lines sharing HLA alleles with patient 8 were pulsed with the minimum NY-ESO-1_88–96_ peptide, extensively washed, co-cultured with NY-ESO-1_88–96_-specific T_CD8+_ line and followed with ICS.

In order to examine the T cell mediated immune response to NY-ESO-1, a systematic 18 mer peptide screen was performed using peripheral blood mononuclear cells (PBMCs) from melanoma patient 8 previously vaccinated with NY-ESO-1 ISCOMATRIX™ vaccine [6]. To preserve PBMC samples, pooled 18 mer peptides were used to stimulate NY-ESO-1 specific T cells in the PBMCs. The cultures containing amplified NY-ESO-1-specific T_CD8+_ were subsequently assessed with individual 18 mer peptides. Our screen showed that the patient had an immunodominant response to NY-ESO-1 in the 79–96 region (Figure 1A). Using overlapping 13mer and shorter peptides within the 79–96 sequence in conjunction with peptide prediction algorithms, we narrowed down the likely minimum epitope to 88–96. To confirm this, the NY-ESO-1_88–96_ peptide and two other shorter peptides were synthesized and tested by the antigen-specific T cell line. As shown in Figure 1B, at 10^−8 ^M and in the absence of serum proteases, NY-ESO-1_88–96_ was able to activate the antigen-specific T_CD8+_, but the two peptides with a single amino acid truncation at either end failed to do so, indicating that NY-ESO-1_88–96_ is the minimal epitope. Typically 50% of specific T cells within such a T_CD8+_ line would be activated by as little NY-ESO-1_88–96_ peptide as 10^−9 ^M (Figure. 1C).

To characterize which HLA class I allele bound the NY-ESO-1_88–96_ peptide, homozygous lymphoblastoid cell lines (LCL) sharing HLA molecule with patient 8, namely 9010, 9039, and 9063, were pulsed with NY-ESO-1_88–96_, washed, and co-cultured with T_CD8+_ specific for NY-ESO-1_88–96_. Clearly these T_CD8+_ produced IFN-γ only when co-cultured with peptide pulsed LCL 9039 (Figure. 1D), which shared HLA-B18 and HLA-Cw5 with patient 8. However, LCL 9063 also expressed HLA-Cw5 and was not able to present NY-ESO-1_88–96_. We further excluded HLA-Cw7 as the restriction allele using NY-ESO-1_88–96_-pulsed LCL that only shared HLA-Cw7 with the patient, which did not activate the T_CD8+_ line (data not shown), and concluded that HLA-B*1801 presented NY-ESO-1_88–96_.

**Figure 2:**
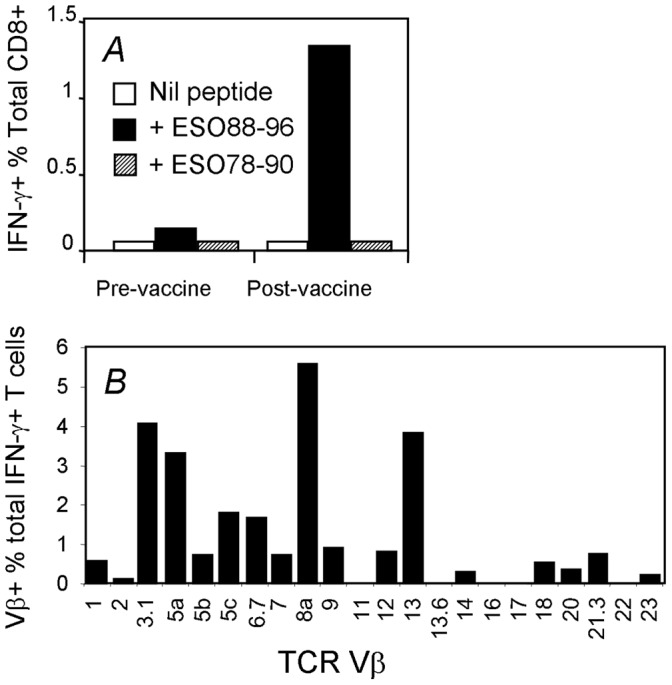
NY-ESO-1_88–96_-specific T cells are vaccine boosted and utilize polyclonal T cell receptors. PBMCs from patient 8 collected before (day 0) and after (day 70) vaccination with NY-ESO-1 ISCOMATRIX™ vaccine were expanded with 18 mer NY-ESO-1_79–96_ and the T cells were assessed by ICS (A). A similar T cell line expanded from day 70 PBMC sample from patient 8 was first stimulated with NY-ESO-1_88–96_ peptide, then split into multiple wells and stained with anti-CD8 and a panel of antibodies specific to various TCR Vβ families separately, followed with ICS for IFN-γ (B). Graph indicates the percentage of NY-ESO-1_88–96_-specific (IFN-γ-producing) T cells expressing the indicated TCR Vβ families.

Having shown that the NY-ESO-1_88–96_-specific T_CD8+_ response is HLA-B18-restricted, and is immunodominant in patient 8, we investigated whether this response was induced by vaccination. We expanded T cells from PBMC of patient 8 collected before or 70 days after vaccination. Although the response to HLA-B18/NY-ESO-1_88–96_ was detectable in the pre-vaccination PBMC sample (0.19% of total T_CD8+_, Figure 2A), it was obviously boosted by the NY-ESO-1 ISCOMATRIX™ vaccine (1.38% of total T_CD8+_ post vaccination, Figure 2A). We then examined whether a similar response can be found in PBMCs from other melanoma patients who also express HLA-B18 and had anti-NY-ESO-1 antibody response, which is often associated with anti-NY-ESO-1 T cell response [27]. Antigen-specific T_CD8+_ were expanded using NY-ESO-1_79–91_ peptide and then assessed their antigen-specificity using both tetramer and intracellular cytokine staining (ICS) for IFN-γ. As shown in Table 1, among eight other HLA-B18^+^ patients, three of the patients had detectable T_CD8+_ specific for HLA-B18/NY-ESO-1_88–96_. Interestingly, all three were incidentally placed in the vaccine groups and the responses were pre-existed before vaccination, indicating that the response we detected in patient 8 is not a single case and the anti-NY-ESO-1_88–96_ response is likely the immunodominant response associated with HLA-B18. However, the other five samples showed no detectable response to this epitope.

**Table 1:** T_CD8+_ response to HLA-B18/NY-ESO-1_88–96_.

Group	Patients	HLA			Tetramer or ICS (% of total CD8+ T cells)		Vaccination status
		HLA-A	HLA-B	HLA-C	Pre-vac	Post-vac	
NY-ESO-1 ISCOMATRIX™Vaccine	8	A68	B1801, B4402	Cw5, Cw7	0.19	1.220	Boosted
	111	A2, A3	B1801, B5101	Cw1,Cw7	2.87	3.76	Pre
	102	A3, A11	B1801, B4403	Cw7,Cw16	3.89	4.76	Pre
	109	A2	B1801, B4402	Cw5,Cw7	0.140	0.140	Pre
	12	A25, A68	B1801, B1402	Cw7,Cw8	ND	–*	
Placebo controls	115	A2, A30	B1801, B3901	Cw5	ND	–*	
	114	A2, A25	B1801, B3701	Cw6	ND	–*	
	29	A1, A31	B18, B27	Cw2,Cw6	ND	–*	
	113	A3, A25	B18, B7	Cw7,	ND	–*	

Melanoma patients from three clinical trials (see Materials and Methods) with detectable anti-NY-ESO-1 antibody responses and HLA-B18 expression were selected for the screen. T cells from PBMC samples post vaccination (or placebo controls that did not receive the NY-ESO-1 ISCOMATRIX™ vaccine but received diluents) were expanded with 18 mer NY-ESO-1_79–96_ peptide for 12∼15 days and assessed with NY-ESO-1_88–96_ in an ICS assay (only ICS results <0.1% are shown as negative “–” indicated by ‘*’). For patients who showed positive T_CD8+_ response to this epitope (>0.1%, data not shown) in their post vaccination samples, pre- and post-vaccination PBMC samples were then expanded the same way side-by-side in a second screen intended to determine whether the response was a result of the vaccination. The peptide-specific T_CD8+_ in the second screen were assessed with the specific HLA-B18/NY-ESO-1_88–96_ tetramer. Tetramer results >0.1% of total CD8^+^ T cells with a discrete staining pattern are shown; and those results <0.1% are shown as “-”. Pre – pre-existed response; Boosted – vaccine-boosted response; ND – not determined, Pre-vac, prior to vaccination; Post-vac, after vaccination.

To further characterize this immunodominant T cell response, we conducted a T cell repertoire analysis on T_CD8+_ specific to HLA-B18/NY-ESO-1_88–96_ in PBMCs from patient 8. While the majority of T cells used Vβ3.1, 5a, 8a and 13, other Vβ chains were also used by the antigen-specific T_CD8+_ (Figure 2B), indicating that the NY-ESO-1_88–96_-specific T_CD8+_ lines used in this study are of polyclonal nature.

### NY-ESO-1_88–96_-specific T_CD8+_ do not Recognize Melanoma Cell Line SK-MEL-8

**Figure 3:**
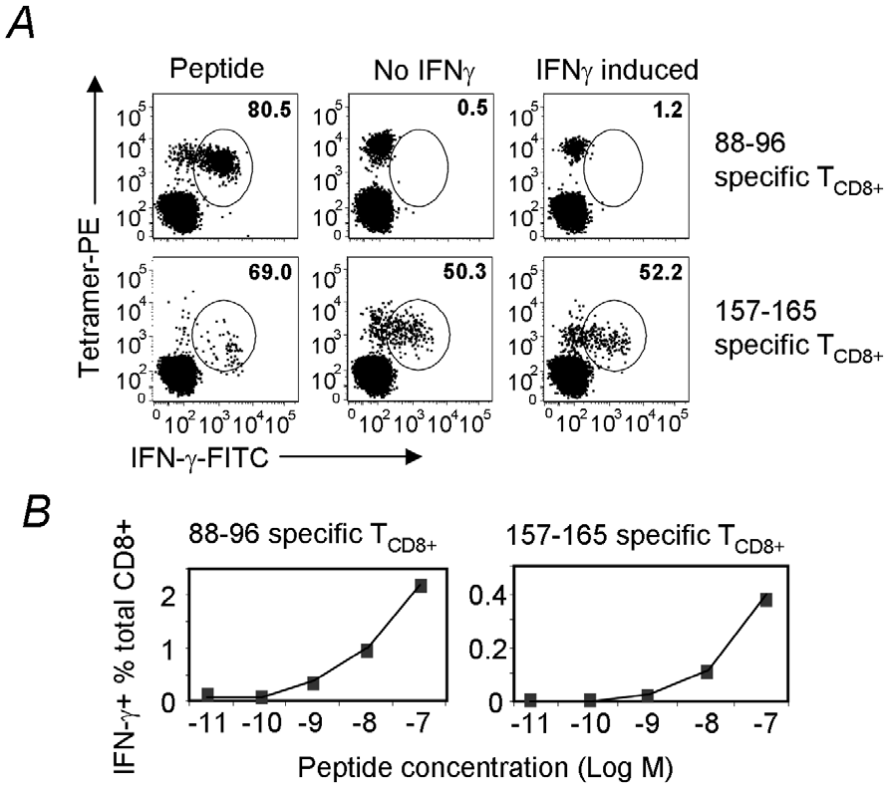
NY-ESO-1_88–96_ is not naturally presented by melanoma cells. *A*, NY-ESO-1_157–165_– and NY-ESO-1_88–96_–specific T_CD8+_ lines were expanded from PBMCs collected from the previously reported patient 7 [6] and patient 8 with 18 mer peptides NY-ESO-1_157–174_ and NY-ESO-1_79–96_ respectively. These T cells were then co-incubated with tumor line (SK-MEL-8) with or without a 48 hr IFN-γ induction (see **Materials and Methods** for details). The untreated SK-MEL-8 cells were also pulsed with both peptides followed by washing out excessive peptides to serve as a maximum antigen presentation control. Antigen-specific T cell activation was then revealed by tetramer and IFN-γ double staining. Percentage represents antigen-specific, IFN-γ-producing cells amongst total tetramer positive cells (note, the double negative cell population was not included in the percentage calculation). *B*, the same T_CD8+_ lines used in A were also assessed for their affinity by peptide titration. Percentage represents Ag-specific T cells among total CD8^+^ T cells. Similar data were obtained from three similar experiments.

We have so far shown that the NY-ESO-1_88–96_-specific T_CD8+_ in most patients were spontaneously generated and can be boosted by vaccination with NY-ESO-1 ISCOMATRIX™ vaccine. It is important to find out whether these T_CD8+_ are able to recognize tumor cells. To this end, we expanded T_CD8+_ lines either specific to HLA-B18/NY-ESO-1_88–96_, or HLA-A2/NY-ESO-1_157–165_, a well characterized and naturally presented epitope to serve as a positive control [3], [29]. The two T_CD8+_ lines were then used to detect direct antigen presentation on a melanoma cell line, SK-MEL-8, which expresses NY-ESO-1 as well as HLA-B18 and HLA-A2. HLA-B18/NY-ESO-1_88–96_ and HLA-A2/NY-ESO-1_157–165_ tetramers were used in combination with ICS for IFN-γ to positively identify the antigen-specific T_CD8+_ [30]. As expected, peptide-pulsed HLA-B18^+^ and HLA-A2^+^ LCL line 9039 (not shown) and SK-MEL-8 induced IFN-γ production from most of the tetramer^+^ T_CD8+_ specific to either epitope (Figure. 3A left panels). Importantly, more than half (50.3% and 52.2%) of the NY-ESO-1_157–165_ specific T_CD8+_ were stimulated to produce IFN-γ by co-culture with SK-MEL-8 tumor cells regardless of the 48 hour IFN-γ induction (Figure. 3A middle and right panels). However, NY-ESO-1_88–96_ specific T_CD8+_ did not respond to the same APCs (Figure. 3A top panels), despite higher avidity for HLA-B18/NY-ESO-1_88–96_ compared to the T_CD8+_ specific to HLA-A2/NY-ESO-1_157–165_ (Figure. 3B), indicating that either HLA-B18/NY-ESO-1_88–96_ is not directly presented or not presented in sufficient quantity by this tumor line, as required to trigger the antigen-specific T_CD8+_.

### Presentation of NY-ESO-1_88–96_ is Immunoproteasome-dependent and Requires Higher NY-ESO-1 Expression

It is well established that IFN-γ treatment of cell lines lead to enhanced antigen processing and presentation, as IFN-γ up-regulates MHC class I expression and switches constitutive proteasome to immunoproteasome [31].

**Figure 4:**
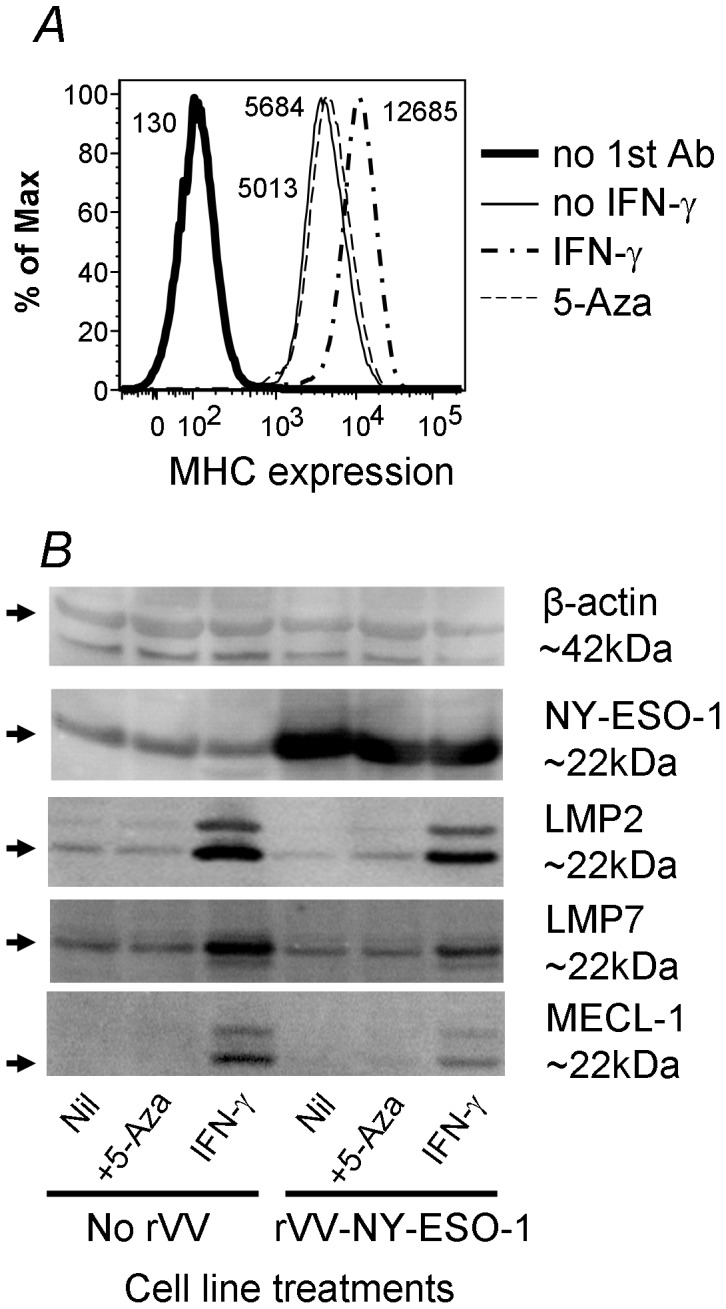
IFN-γ-induction induces immunoproteasome and rVV-NY-ESO-1 infection significantly increases NY-ESO-1 expression in tumor cells. Tumor cell line, SK-MEL-8, was treated with either IFN-γ for 48 hrs or 5-aza-dC for 72 hrs. The cells were then stained for their surface class I expression (A). The treated cells were also infected with rVV-NY-ESO-1, or rVV-GFP as a control, for 4 hours. The infected and uninfected cells were then lysed immediately for Western analysis (B). Similar data were obtained from three similar experiments. Note, β-actin was reblotted after stripping the previously bound anti-NY-ESO-1 antibody.

The apparent lack of endogenous presentation of HLA-B18/NY-ESO-1_88–96_ by this tumor cell line was further investigated, in combination with IFN-γ treatment, using the following two strategies to enhance NY-ESO-1 expression. Firstly, we treated tumor line SK-MEL-8 with DNA hypomethylation agent 5-aza-2-deoxycytidine (5-aza-dC), which has been reported to upregulate cell surface class I and induce or upregulate the expression of different CT antigens in cultured human melanoma lines [32]. Secondly, we infected the cell line with recombinant vaccinia viruses encoding NY-ESO-1 (rVV-NY-ESO-1), which was expected to boost NY-ESO-1 expression. As shown in Figure 4A, the IFN-γ treatment increased cell surface class I expression by two fold for SK-MEL-8. However, 5-aza-dC treatment did not increase class I expression. We also performed the treatment under various concentration of 5-aza-dC and similar results were obtained (data not shown). We next used Western blotting to assess the changes in the expression of NY-ESO-1 and the immunoproteasome subunits for the tumor cell line after these treatments. As shown in Figure 4B, SK-MEL-8 expressed NY-ESO-1 at a level that was readily detected and was not further induced by 5-aza-dC treatment. IFN-γ treatment did not enhance NY-ESO-1 expression although it clearly induced the expression of the immunoproteasome subunit LMP2, LMP7 and MECL-1 (Figure 4B) indicating the switching over from constitutive proteasome to immunoproteasome. Of note, there are faint, similar sized bands revealed by both anti-LMP2 and anti-LMP7 anti-sera before IFN-γ treatment. It is possible that these cells may express low level of these subunits under normal culture condition. As expected, rVV-NY-ESO-1 infection of the tumor line greatly boosted NY-ESO-1 expression (∼6-fold increase according to the analysis performed using ImageQuant TL software, Amershan Biosciences, data not shown). Importantly, the above-described changes were treatment-specific because the internal loading control of each sample, β-actin, remained unchanged (Figure 4B top and data not shown).

**Figure 5:**
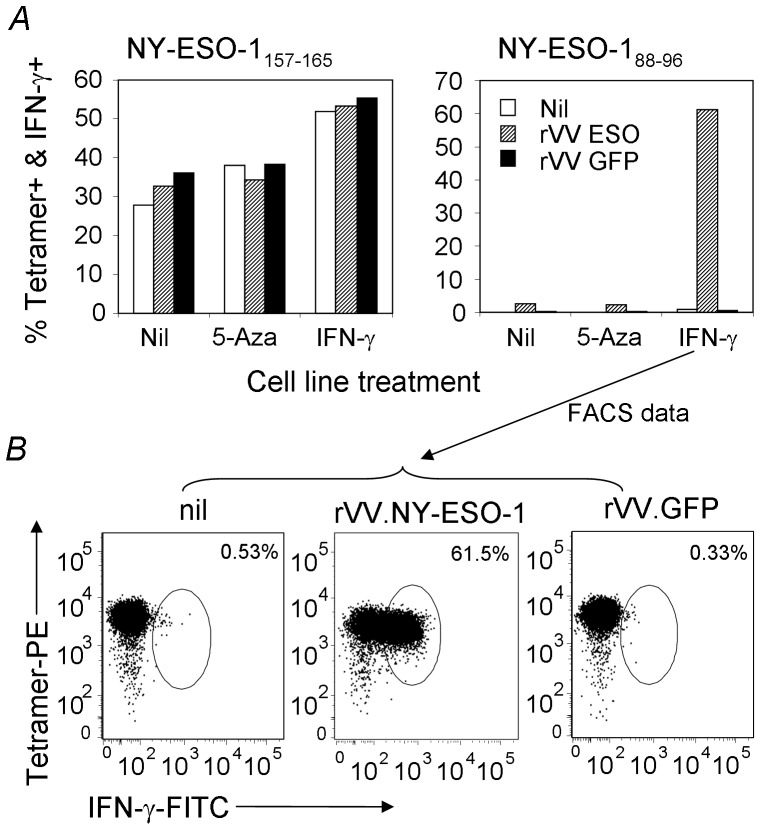
NY-ESO-1_88–96_ is poorly presented directly by tumor cells. In A and B, Tumor cells were treated as described in Figure 4. The treated tumor cells were then co-cultured with T cell lines specific for NY-ESO-1_157–174_ and NY-ESO-1_88–96_ as described in Figure 3. The purity of the T_CD8+_ lines were >70% (data not shown). The antigen presentation results are shown in A and the FACS plots corresponding to NY-ESO-1_88–96_ T_CD8+_ responses to IFN-γ treated SK-Mel-8 are shown in B (shown by the arrow). Similar data were obtained from three similar experiments.

Using similar T cell lines as shown in Figure 3, we tested the above treated SK-MEL-8 cells for their antigen presenting capacity. NY-ESO-1_157–165_ was again efficiently presented without the treatments. However, the IFN-γ-treated SK-MEL-8 showed enhanced antigen-presenting capacity for NY-ESO-1_157–165_ (Figure 5A). By contrast, the HLA-B18/NY-ESO-1_88–96_ specific T_CD8+_ were not activated by the same cells, unless they were both IFN-γ-treated and rVV-NY-ESO-1 infected (Figure 5A and the FACS plots in Figure 5B correspond to IFN-γ-induced SK-MEL-8 that were either uninfected or infected with rVV-NY-ESO-1 or rVV-GFP). These data imply that the NY-ESO-1_88–96_ epitope was processed inefficiently from endogenous antigen and the processing relied on the action of the immunoproteasome rather than the constitutive proteasome.

### NY-ESO-1_88–96_ is Directly Presented by Tumor Cells Expressing High Level of Immunoproteasome

**Figure 6:**
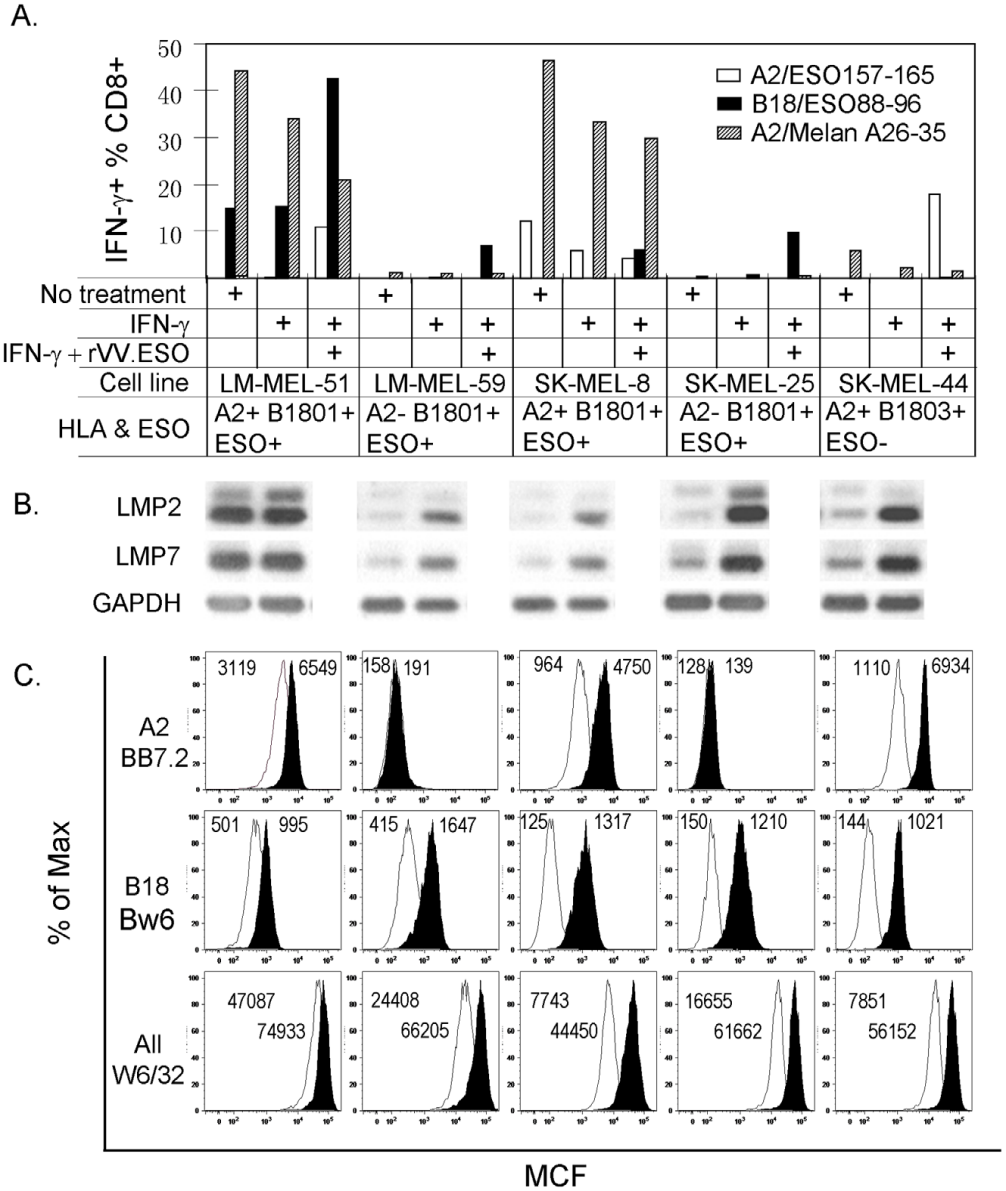
NY-ESO-1_88–96_ is directly presented by tumor cells expressing high level of immunoproteasome. Five melanoma lines, including SK-MEL-8, were left untreated or treated with either IFN-γ for 48 hrs or were further infected with rVV.NY-ESO-1 for 5 hrs. The tumor cells were then co-cultured with T cell lines specific for NY-ESO-1_157–174_, NY-ESO-1_88–96_ and Melan A26–35 as described in Figure 3. The purity of the T_CD8+_ lines were 42%, 88% and 68% respectively (data not shown). The antigen presentation results are shown in A and the western blot results for LMP2, LMP7 and the loading control GAPDH for the corresponding tumor lines and the treatment conditions are shown in B. The FACS analysis results of the cell surface HLA molecules as Mean Channel Fluorescence intensity (MCF) are shown in C. The MCF values for HLA-A2 and B18 were about 100 for the FITC-conjugated secondary antibody alone; and those values for the All Class I group for the PE-conjugated secondary antibody alone were about 300. Similar data were obtained from three similar experiments.

To exclude the possibility that the above results were the biased outcome of a single melanoma line SK-MEL-8, we collected four other HLA-B18 expressing melanoma lines (for detailed HLA class I typing information, please see Table S1). These lines were again left untreated or treated with IFN-γ to induce both immunoproteasome and cell surface class I expression; or after IFN-γ-induction the cells were further infected with rVV-NY-ESO-1 to either induce or enhance NY-ESO-1 expression. As IFN-γ can induce many changes in gene expression, the changes of the immunoproteasome in these cell lines were further monitored functionally by the change of antigen presentation for the HLA-A2/Melan A26–35 epitope as its presentation was reported previously to be impaired by the immunoproteasome [15]. As shown in Figure 6A, although LM-MEL-59, SK-MEL-8 and SK-MEL-25 all expressed NY-ESO-1 antigen and B*1801, they did not present NY-ESO-1_88–96_, unless they are both IFN-γ-induced and infected with rVV-NY-ESO-1. This recognition also critically depends on the high avidity of NY-ESO-1_88–96_-specific T cell line as a lower avidity line failed to do so (Figure S1A, B). The recognition is not an artifact of rVV infection as the same tumor line transiently transfected with pcDNA3-NY-ESO-1 during IFN-γ-induction also presented this epitope (Figure S1C). However, SK-MEL-44 was not able to present NY-ESO-1_88–96_, even under the same treatment regime with similar HLA expression level. It is highly likely that the single AA substitution in B*1803 (Y74D) from B*1801, which is located on the α1-helixes of the peptide-binding cleft, had either prevented NY-ESO-1_88–96_ from binding B*1803 or the T cells from recognizing B*1803/NY-ESO-1_88–96_.

Overall, most tested melanoma cell lines expressed detectable immunoproteasome subunit LMP2 and LMP7 although at relatively low level (Figure 6B). Interestingly, the cell line LM-MEL-51 presented NY-ESO-1_88–96_ quite efficiently even when it was not treated with IFN-γ although rVV-NY-ESO-1 infection did further enhance the presentation. However, this cell line expressed high level of LMP2 and LMP7 under normal cell culture conditions and the IFN-γ induction did not further induce such expression significantly (Figure 6B). Of note, the immunoproteasome expression level in LM-MEL-51 in the absence of IFN-γ-induction seemed to be even higher than that of other lines after IFN-γ-induction. Importantly, as an internal control the presentation of Melan A_26–35_ by the three HLA-A2 positive cell lines were easily detected and decreased after IFN-γ-induction, confirming their impaired presentation by the immunoproteasome reported by Morel et al [15] and further implying the induction of the immunoproteasome by IFN-γ treatment. It is not clear why LM-MEL-51 did not present NY-ESO-1_157–165_ as well as SK-MEL-8 although the former expressed higher level of HLA-A2 (Figure 6C and Figure S1B). It is possible that the direct presentation of NY-ESO-1_157–165_ requires the activity of the constitutive proteasome, perhaps even more so than that required by the Melan A_26–35_. Taken together, the direct presentation of NY-ESO-1_88–96_ by melanoma lines requires high level of both immunoproteasome and NY-ESO-1 expression by tumor lines.

### NY-ESO-1_88–96_ is Efficiently Cross-presented by MoDC

**Figure 7:**
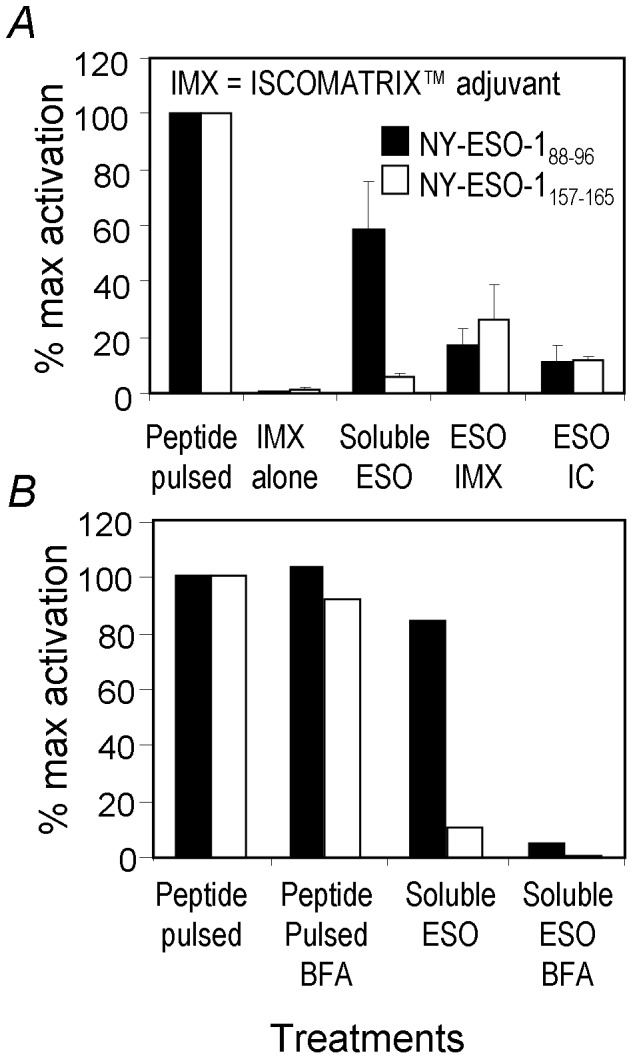
NY-ESO-1_88–96_ is cross-presented efficiently by DCs from soluble antigen. In A, MoDCs expressing both HLA-A2 and HLA-B18 were cultured for 7 days, and then incubated overnight under the indicated conditions before being co-cultured with the indicated T_CD8+_ lines for 5 hrs in the presence of BFA. NY-ESO-1 specific T_CD8+_ activation was assessed by tetramer and ICS. IFN-γ producing cells out of total antigen-specific (tetramer positive) T_CD8+_ were converted to percentages of maximum activation induced by the respective minimum peptide (peptide activation of NY-ESO-1_157–174_ T_CD8+_ line and NY-ESO-1_79–96_ T_CD8+_ line were both 30% to 45% for all three experiments conducted, data not shown) and plotted as “% Maximum activation”. After data conversion, mean values and standard deviations were calculated from data obtained from three similar experiments. In B, one of the control experiments was shown for APCs that were either pulsed with the corresponding peptide or soluble NY-ESO-1 for one hour followed with BFA addition to demonstrate the nature of intracellular cross-presentation for both T_CD8+_ epitopes without affecting extracellular peptide presentation. Similar results were obtained twice.

Knowing that the HLA-B18/NY-ESO-1_88–96_ response is often detectable in melanoma patients and that anti-tumor immunity relies on cross-presentation, we hypothesized that this epitope is efficiently cross-presented by DCs. Our previous studies have shown that the antigen formulations influence the efficiency and the pathways of antigen processing and cross-presentation [3], [21], [28]. To investigate the cross-presentation of NY-ESO-1_88–96_ from full-length antigen, MoDCs expressing both HLA-B*1801 and HLA-A*0201 were incubated overnight in the presence of different NY-ESO-1 antigen formulations including soluble NY-ESO-1 protein, NY-ESO-1 ISCOMATRIX™ vaccine and NY-ESO-1/IC and subsequently co-cultured with T_CD8+_ lines specific to either HLA-A2/NY-ESO-1_157–165_ or HLA-B18/NY-ESO-1_88–96_. T_CD8+_ activation was again enumerated with tetramer and ICS. As shown in Figure 7A, 15 to 30% of NY-ESO-1-specific T_CD8+_ were activated by NY-ESO-1/IC and NY-ESO-1 ISCOMATRIX™ vaccine, respectively for the two T_CD8+_ lines, although the NY-ESO-1_157–165_ epitope was cross-presented slightly more efficiently than NY-ESO-1_88–96_. Few T cells of the T_CD8+_ line specific to HLA-A2/NY-ESO-1_157–165_ were activated by the MoDCs incubated with soluble NY-ESO-1 protein. By contrast more than 60% of NY-ESO-1_88–96_ specific T_CD8+_ were activated by the same MoDCs incubated with the soluble NY-ESO-1 protein. We further demonstrated that these MoDCs first took up the soluble NY-ESO-1 protein followed by endogenous antigen processing to present these epitopes; because under the same conditions with BFA addition, which prevents the HLA from leaving the endoplasm reticulum, cross presentation was abolished (Figure 7B). This indicated that in our system the NY-ESO-1_88–96_ peptide was generated intracellularly after antigen uptake and not extracellularly by serum proteases. We attempted incubating NY-ESO-1^+^ tumor cell lysates with MoDCs in such assays with no success (data not shown). We believe that the NY-ESO-1 protein amount might be too little relative to the total cellular protein amount in the tumor lysates.

## Discussion

In the process of monitoring T cell responses induced by the NY-ESO-1 ISCOMATRIX™ vaccine, we identified a novel NY-ESO-1_88–96_ epitope presented by HLA-B18 molecule. The polyclonal T_CD8+_ response to this epitope was immunodominant in PBMC from patient 8 and was clearly boosted by our vaccination. The NY-ESO-1_88–96_ peptide was efficiently cross-presented by MoDCs pulsed with soluble, recombinant NY-ESO-1 protein, which is different to what has been found for other NY-ESO-1-derived T_CD8+_ epitopes [3], [21], [28].

It is well established that direct antigen presentation typically requires ongoing antigen synthesis and proteasome-mediated degradation [16], [17]. However, cross-presentation does not require sustained antigen synthesis and degradation in antigen-donating cells [19]. Given these contrasting requirements between the two presentation pathways and taking into account the peptide cleavage preferences for the constitutive proteasome and the immunoproteasome respectively, there has been surprisingly no reported example demonstrating differential cross- and direct-presentation in human APC. Ochsenbein et al [33] showed that, in an artificial tumor antigen system in mice, there was direct priming in the absence of detectable cross-priming for the LCMV-derived GP33 epitope expressed on either fibroblasts or EL-4 thymoma cells. In this system, syngeneic tumor cells were used and it was not clear whether or not priming relied on cross-presentation by DCs. Consequently, these authors questioned the physiological significance of cross-presentation in anti-tumor and anti-viral immune responses [33]. In an HLA-A2 transgenic mouse model, Chapatte et al showed that only immunoproteasome deficient DCs were able to prime T_CD8+_ response to a melanoma differentiation antigen Melan A [34]. However, in this system DCs were infected with a non-replicative lentivirus expressing Melan A before being transferred into naïve mice. Therefore, potentially only direct antigen presentation and priming were assessed, which is likely not the major role DCs play in anti-tumor immunity [34]. Our results clearly demonstrated that cross-presentation of tumor antigens, such as NY-ESO-1_88–96_, occurs readily in cultured MoDCs, especially from soluble form of NY-ESO-1. We are not aware of any other T cell epitope that is more efficiently cross-presented from soluble antigen than complexed antigen forms. This is likely also the case *in vivo,* because among the nine melanoma patients screened in our study, four had detectable HLA-B18/NY-ESO-1_88–96_ responses, while there was no direct presentation of this epitope by most melanoma cells tested. The same melanoma cells were able to present NY-ESO-1_157–165_ efficiently under the same conditions (Figure 5, 6, and Figure S1B, C) indicating that the amount of NY-ESO-1 expressed by the tumor lines is physiologically sufficient and the antigen processing and presentation machinery is normal. Judging from the NY-ESO-1 amount after rVV-NY-ESO-1 infection (Figure 4B), with more than 6-fold increase in expression in the infected SK-MEL-8, it is inconceivable that such level of NY-ESO-1 expression might be achieved *in vivo* under physiological conditions. Therefore, it is difficult to envisage that the HLA-B18/NY-ESO-1_88–96_ epitope would be ever presented in sufficient level directly on tumor cell surface unless there was a concomitant infection in the tumor-bearing host and the infection resulted in sufficient IFN-γ production to induce immunoproteasome in these cells. Interestingly, one of the melanoma lines tested, LM-MEL-51, expressed high level of immunoproteasome and directly presented NY-ESO-1_88–96_ (Figure 6A). However, it is not clear whether such property was developed *in vivo* or *in vitro*. These results not only indicate that the two NY-ESO-1-derived epitopes are processed and presented differently by the intracellular antigen processing and presentation machinery, for both direct- and cross-presentation; but also provide strong evidence that some of the epitopes may not serve as direct target on tumor cells.

In patients with such T cell responses, the tumor cells would therefore behave as natural immune escape mutants, much the same as those that have down regulated their MHC class I expression and lost their antigen-presentation capacity [22], [35]. However, the key difference here maybe that the antigen is likely efficiently taken up by tumor stroma cells (including DCs, macrophages and perhaps others) and the T_CD8+_ epitopes are then more efficiently displayed. It is not yet known whether stroma elimination by such T_CD8+_ would be beneficial in humans. However, Spiotto et al. recently demonstrated that murine tumor cells lacking antigen-presenting MHC molecules were controlled by T_CD8+_ specific for antigens expressed by these tumor cells through the elimination of stroma cells that cross-presented the T cell epitopes from the same tumor antigens [36]. Tumor stroma has been known to play a major role to support tumor growth and sometimes to suppress the immune system [37]. It is therefore possible that T_CD8+_ specific for epitopes that are efficiently cross-presented might actually play an important role, albeit indirect, to keep tumors in check.

On the other hand, if such T_CD8+_ responses are immunodominant, such is the case in our patient 8, the subdominant yet tumor-recognizing T_CD8+_ responses may not be efficiently stimulated due to excess expansion of the immunodominant T_CD8+_ and, as a result of that, elimination of the cross-presenting DCs. Therefore, T_CD8+_ with this specificity will further expand and prevent other potentially more beneficial, but subdominant T_CD8+_ responses from being activated or expanded by the same DCs, a phenomenon termed as immunodomination [23]. There is an example in immunity against influenza A virus in C57BL/6 mouse model, in which vaccinating T_CD8+_ specific to the immunodominant epitope from acidic polymerase PA_224–233_ caused delayed viral clearance, because this epitope is only generated by DCs [38], [39] due to its strict immunoproteasome dependence [13], [14].

Biased differential antigen presentation by either the immunoproteasome or the constitutive proteasome has been reported for directly presented, tumor derived epitopes [15], [34], [40], [41]. It has also been recently shown that human MoDCs were more capable of presenting T cell epitopes from melanoma antigens when immunoproteasome expression was knocked down by small interference RNA (siRNA) [42]. However, the later study investigated T cell responses in already ‘primed’ melanoma patients and did not directly address the role of DC’s cross-presentation in anti-tumor immunity, because the tumor antigens studied were all introduced into these DCs by RNA transfection [42]. Our study demonstrated a near ‘black-and-white’ outcome between direct- and cross-presentation for the NY-ESO-1_88–96_ epitope unless high level immunoproteasome is expressed by tumor cells. Importantly, our results may indicate the existence of a whole group of T_CD8+_ epitopes resulted from differential antigen processing and presentation between DCs and tumor cells. As NY-ESO-1_88–96_-specific T cells are naturally primed and immunodominant in the melanoma patient 8 examined in detail in our study, our findings have important implications for future vaccine design. For example, it might be desired to avoid priming or boosting such T_CD8+_ by using mutated NY-ESO-1. For instance mutating the anchor residues (89E or 96F) to an Alanine for the NY-ESO-1_88–96_ epitope would abrogate the stimulation of its specific T_CD8+_. Alternatively, using an antigen form rather than the soluble one may minimize the stimulation to these T_CD8+_ if their expansion results in immunodomination. Conversely, if these T_CD8+_ play a positive role through the elimination of stroma cells, as reported in the above mentioned murine system, vaccination with either the soluble form of NY-ESO-1 or the minimal peptide NY-ESO-1_88–96_ would then be ideal. Therefore, using full-length tumor antigen as vaccine, although potentially providing broader coverage for T cell epitopes and HLA polymorphism as it may provide all the available epitopes, could be an over-simplified strategy due to the lack of consideration on differential direct- and cross-presentation, not to mention it does not avoid stimulating potential antigen-specific regulatory T cells [43].

## Materials and Methods

### Patients

Melanoma patients (listed in Table 1) were vaccinated by intramuscular injection with 100 µg NY-ESO-1 ISCOMATRIX™ vaccine from LUD99-008 [6] or LUD2002-013 (ClinicalTrials.gov Identifier: NCT00518206) and LUD2003–013 [44]. The LUD99-008 study included patients who received placebo or NY-ESO-1 protein alone and showed that these cohorts were not effectively vaccinated. All studies were approved by the Human Research Ethics Committees of Austin Health and the Peter MacCallum Cancer Center. All patients provided written informed consent.

### Peptides, Antibodies, Tetramers and NY-ESO-1 Formulations

NY-ESO-1 overlapping 18 mers, 9mers (88–96 (LEFYLAMPF) and 157–165 (SLLMWITQC)) and shorter peptides and Melan A26–35 (EAAGIGILTV) were synthesized by Auspep (Melbourne, Australia). Monoclonal antibodies to CD8, CD4, IFN-γ and HLA class I (W6/32) were purchased from BD (Franklin Lakes, NJ) and the anti-HLA-A2 (BB7.2), Bw6 (which recognizes B18 and a few other HLA-B alleles) were used as hybridoma culture supernatants. PE-conjugated HLA-B18/NY-ESO-1_88–96_ and HLA-A2/NY-ESO-1_157–165_ tetramers were synthesized at the Tetramer Production Facility of the Ludwig Institute for Cancer Research (Lausanne, Switzerland). Flow cytometry was performed using a BD FACSCalibur or FACSCanto II instrument, and data were analyzed using FlowJo software (TreeStar Inc., Ashland, OR).

Full length recombinant NY-ESO-1 protein was produced in *E coli* and purified in the GMP facility of the Ludwig Institute for Cancer Research at the Memorial Sloan-Kettering Cancer Center (New York, USA). Endotoxin levels ranged between 3–31 EU/0.1 mg of protein (limit <175 EU/0.1 mg protein). ISCOMATRIX™ adjuvant (CSL Limited, Victoria, Australia) formulated NY-ESO-1 was generated as described [45], [46]. Immune complexes (ICs) (NY-ESO-1/IC) were generated by mixing NY-ESO-1 protein with anti-NY-ESO-1 mAb ES121 at a 1∶2 molar ratio in serum-free RPMI-1640 at 37°C for 30 min as previously described [3].

### Cell Lines, MoDCs, T Cell Culture and Media

All cell lines are maintained in complete medium RF-10 consisting of RPMI-1640 supplemented with 2 mM Glutamax, antibiotics, 10 mM HEPES (Invitrogen, Carlsbad, CA), 1% non-essential amino acids and 10% fetal calf serum (FCS, Thermo Trace, Melbourne, Australia). The SK-MEL-8, SK-MEL-25 [47] and SK-MEL-44 [48] melanoma lines were obtained from the Memorial Sloan-Kettering Cancer Center. LM-MEL-51 and LM-MEL-59 melanoma cell lines were established in our laboratory from melanoma biopsies. Epstein-Barr virus (EBV) transformed, homozygous LCL 9010, 9063 and 9039 were made available from the 10^th^ International HLA Workshop (New York). PBMCs were prepared from whole blood by Ficoll-Paque centrifugation. T cell lines were generated using RF-10 containing 25 U/ml interleukin-2 (IL-2, Cetus, Emeryville, CA). PBMC from melanoma patients expressing HLA-A2 and HLA-B18 and previously vaccinated with NY-ESO-1 ISCOMATRIX™ vaccine were stimulated with 18 mers NY-ESO-1_79–96_ (GARGPESRLLEFYLAMPF containing NY-ESO-1_88–96_) and NY-ESO-1_157–174_ (SLLMWITQCFLPVFLAQP containing NY-ESO-1_157–165_) at 5×10^−6 ^M for 1 hour at room temperature [49]. T cell lines were used at least 12 days after culture. In some cases, tetramer enriched antigen-specific T cells were further expanded by PHA and allogeneic feeders. T cell specificity was confirmed by tetramer staining. For the 18 mer screen, PBMC from patient 8, post-vaccination (70 days) with NY-ESO-1 ISCOMATRIX™ vaccine, were cultured for 13 days with pooled overlapping NY-ESO-1 18 mer peptides (3 or 4 peptides per pool) and then tested for responsiveness to individual 18 mer peptide within the pool by ICS for IFN-γ [50]. Pre- and post-vaccination PBMCs from patients who received NY-ESO-1 ISCOMATRIX™ vaccine were also tested in parallel to determine whether the B18-restricted, NY-ESO-1_88–96_-specific response was vaccinated or occurred naturally. MoDC were generated from CD14^+^ monocytes enriched by MACS beads (Miltenyi Biotec, Auburn, CA) in the presence of 10 ng/mL IL-4 and 20 ng/mL GM-CSF for 6 days [3].

### IFN-γ and 5-aza-2-deoxycytidine Treatment of Tumor Cell Line

The melanoma cell line was cultured in either RF-10 or RF-10 plus 50 ng/ml recombinant human IFN-γ (PeproTech) for 48 hrs before being used as APCs. 5-aza-2-deoxycytidine (5-aza-dC) was purchased from Sigma-Aldrich. Melanoma cells were pulsed with 0.5 µM 5-aza-dC every 24 h for 3 days as previously described [32], then used for the indicated experiments and for FACS assessment of their surface Class I molecules using mouse monoclonal antibodies (as hybridoma culture supernatants) BB7.2 (HLA-A2), Bw6 (B18) and W6/32 (pan-class I molecules). FITC conjugated secondary antibody was used for BB7.2 and Bw6 and PE-conjugated secondary antibody was used for W6/32 readout.

### Antigen Pulsing and Recombinant Vaccinia Virus Infections

For peptide pulsing, cells were incubated with 10^−6^ M peptide for 1 hour at room temperature, washed extensively before being incubated with specific T_CD8+_. Recombinant vaccinia virus (rVV) encoding NY-ESO-1 (rVV-NY-ESO-1) or Green Fluorescent Protein (rVV-GFP) were gifts from Dr. Lloyd Old (Ludwig Institute for Cancer Research, New York, USA) and Drs Jonathan Yewdell and Jack Bennink (National Institute of Health, Bethesda, Maryland, USA), respectively. Cells were incubated with rVV at a multiplicity of infection (MOI) of 10 for 4 hours at 37°C. Infected cells were then incubated with T_CD8+_ lines for antigen presentation readout.

### Western Blotting

Cells were lysed in 1% Triton-X (Sigma-Aldrich), and SDS-PAGE analyses were performed. The proteins were transferred electrophoretically to a polyvinylidene difluoride membrane (Millipore). Separate Western blots were performed for NY-ESO-1, LMP2, LMP7 and MECL-1 expression. All blots used either β-actin or GAPDH as loading control. After transfer, the membranes were incubated with the primary anti-β-actin or GAPDH (abcam) plus anti-NY-ESO-1 mAb (ES121, [50]), or the anti-LMP2 polyclonal rabbit anti-serum (abcam), or the anti-LMP7 polyclonal rabbit anti-serum (abcam), or the anti-MECL-1 polyclonal rabbit anti-serum (BIOMOL) at 4°C overnight. All anti-sera were used at 1∶2000 dilutions. The membranes were washed in PBS, peroxidase-labeled sheep anti-rabbit immunoglobulins or sheep anti-mouse immunoglobulins for NY-ESO-1 (Silenus Labs) were added at a 1/2500 dilution in PBS with 0.05% Tween 20. After further washing, the proteins were visualized radiographically using an electrochemiluminescence (ECL Plus) substrate (Amersham Biosciences) using a STORM phosphoimager.

### T Cell Function Assay

ICS was used in combination with tetramer staining as previously reported by our group [30]. Briefly, cultured T cells were re-stimulated with peptides for 4 hours in the presence of 10 µg/mL Brefeldin A (BFA, Sigma-Aldrich). The cells were then stained with tetramer, anti-CD4 and anti-CD8, fixed with 1% paraformaldehyde (ProSciTech, Queensland, Australia) and further stained with anti-IFN-γ in the presence of 0.2% saponin (Sigma-Aldrich). Up to 30,000 events were recorded on a FACS instrument and analyzed using FlowJo software.

For peptide titrations, 10^5^ cultured T cells were incubated for 4 hours in the presence of 10 µg/mL BFA and serial dilutions of peptide followed by ICS readout. In direct presentation assays, 10^5^ T cells were co-cultured with 5×10^4^ tumor cells for 4 hours in the presence of 10 µg/mL BFA followed by ICS readout. For cross presentation, MoDCs were incubated overnight with 2 µg NY-ESO-1 protein, NY-ESO-1 ISCOMATRIX™ vaccine or NY-ESO-1/IC with 1 µg/mL CD40L-trimer (a kind gift from Amgen) [3], [28], in the presence or absence of 5 µg/mL BFA. Tetramer and IFN-γ double positive cells were used to calculate the percentages of activated, antigen-specific T_CD8+_. Cells pulsed for 1 hour with NY-ESO-1_88–96_ peptide and washed before incubation with T cells served as positive controls. For assessing TCR Vβ usage of peptide-specific T_CD8+_, peptide-expanded T cells from patient 8 were first activated with NY-ESO-1_88–96_ peptide, then split into multiple wells and stained with anti-CD8 and a panel of antibodies specific to various TCR Vβ families separately followed with ICS for IFN-γ.

## Supporting Information

Figure S1 **T_CD8+_ line from patient 102 is of lower avidity and extra NY-ESO-1 expression via transfection also enhances NY-ESO-1_88–96_ presentation.** T cell lines were established using PBMC samples from Patient 8 or 102 under similar conditions. The early cultures were then enriched through tetramer-guided sorting and further expanded using PHA non-specific stimulation. Various tumor lines were either untreated, or treated for 48 hrs with IFN-γ alone, rVV-NY-ESO-1 infected for 5 hrs, or doubly treated with IFN-γ followed by rVV-NY-ESO-1 infection before being used as APC to stimulate T cell lines either specific for A2/NY-ESO-1_157–165_ or B18/NY-ESO-1_88–96_. In A, peptide titration assay was performed by ICS without tetramer staining. The purity of the T cell lines were: patient 8 NY-ESO-1_88–96_ line 88%; patient 102 NY-ESO-1_88–96_ line 42%; and the NY-ESO-1_157–165_ line 66%. B, for the direct presentation, ICS combined with specific tetramer staining was conducted simultaneously as the titration assay shown in A. In C, SK-MEL-8 cells were either untreated, or induce with IFN-γ, or transiently transfected (without selection) with pc3DNA-NY-ESO-1, 5 hrs later induced with IFN-γ for 48 hrs before being used as APC. This was conducted on the same day using the same patient 8 NY-ESO-1_88–96_ T cell line as that in A and B. Similar results were obtained twice.
(TIF)

Table S1 **Melanoma line HLA typing.**
(DOC)
