# Supplementary figures and images for: A Novel HLA-B18 Restricted CD8+ T Cell Epitope Is Efficiently Cross-Presented by Dendritic Cells from Soluble Tumor Antigen
Source: PLoS One. 2012 Sep 6;7(9):e44707. doi: 10.1371/journal.pone.0044707 (PMC3435279; doi:10.1371/journal.pone.0044707)

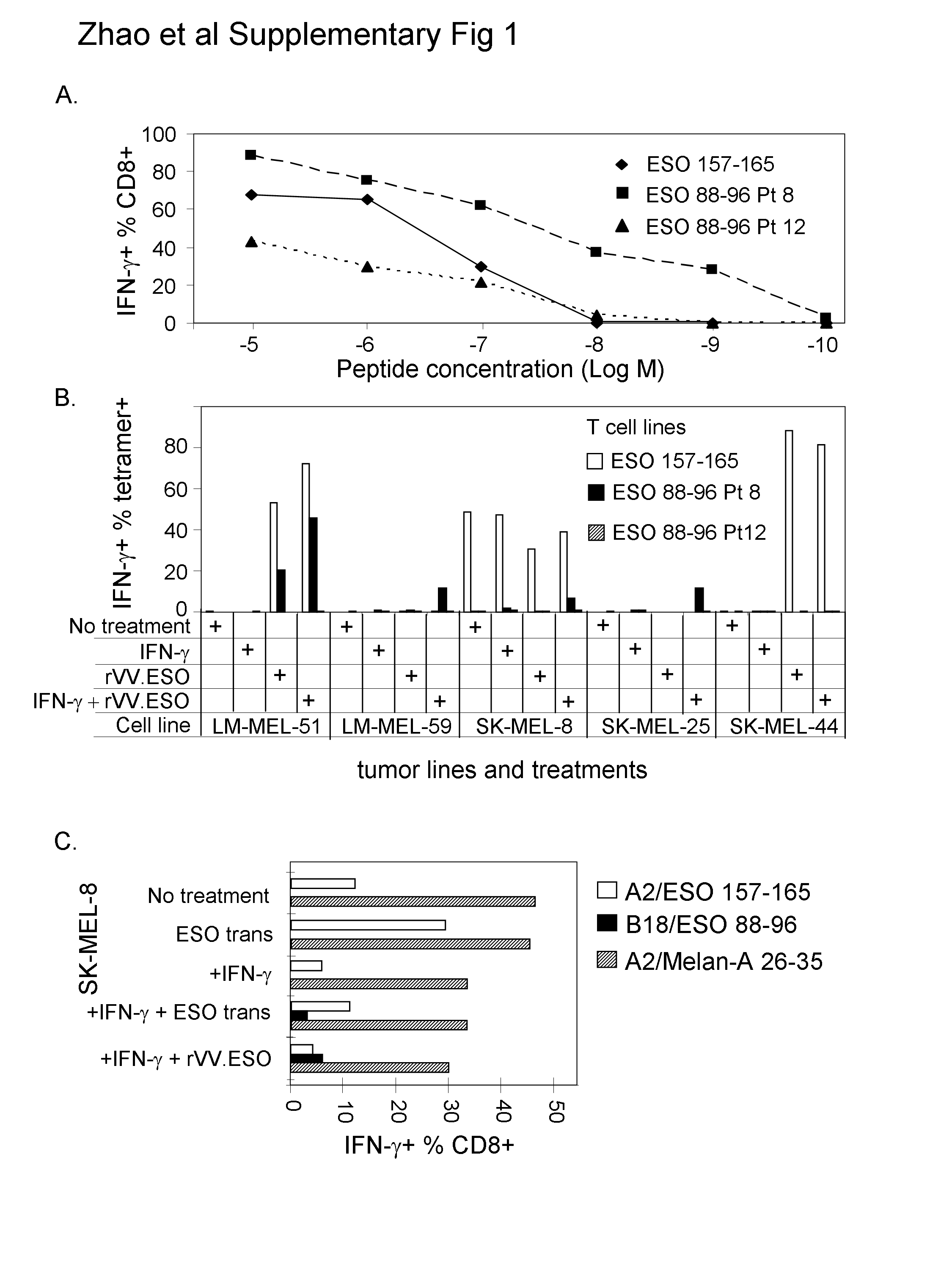

Supplement: Figure S1 — TCD8+ line from patient 102 is of lower avidity and extra NY-ESO-1 expression via transfection also enhances NY-ESO-188–96 presentation. T cell lines were established using PBMC samples from Patient 8 or 102 under similar conditions. The early cultures were then enriched through tetramer-guided sorting and further expanded using PHA non-specific stimulation. Various tumor lines were either untreated, or treated for 48 hrs with IFN-γ alone, rVV-NY-ESO-1 infected for 5 hrs, or doubly treated with IFN-γ followed by rVV-NY-ESO-1 infection before being used as APC to stimulate T cell lines either specific for A2/NY-ESO-1157–165 or B18/NY-ESO-188–96. In A, peptide titration assay was performed by ICS without tetramer staining. The purity of the T cell lines were: patient 8 NY-ESO-188–96 line 88%; patient 102 NY-ESO-188–96 line 42%; and the NY-ESO-1157–165 line 66%. B, for the direct presentation, ICS combined with specific tetramer staining was conducted simultaneously as the titration assay shown in A. In C, SK-MEL-8 cells were either untreated, or induce with IFN-γ, or transiently transfected (without selection) with pc3DNA-NY-ESO-1, 5 hrs later induced with IFN-γ for 48 hrs before being used as APC. This was conducted on the same day using the same patient 8 NY-ESO-188–96 T cell line as that in A and B. Similar results were obtained twice. (TIF) [file pone.0044707.s001.tif]
