# Supplementary material for: A Novel HLA-B18 Restricted CD8+ T Cell Epitope Is Efficiently Cross-Presented by Dendritic Cells from Soluble Tumor Antigen
Source: PLoS One. 2012 Sep 6;7(9):e44707. doi: 10.1371/journal.pone.0044707 (PMC3435279; doi:10.1371/journal.pone.0044707)
Supplement: Table S1 — Melanoma line HLA typing. (DOC) [file pone.0044707.s002.doc]

**Table S**I. Melanoma line HLA typing

| Melanoma lines | HLA-A |  | HLA-B |  | HLA-C |  |
| --- | --- | --- | --- | --- | --- | --- |
| LM-MEL-51 | A0201 | A0301 | B1801 | B5101 | Cw1203 | Cw1402 |
| LM-Mel-59 | A1101 | A2402 | B1801 | B5201 | Cw0701 | Cw1202 |
| SK-MEL-8 | A0201 | A0101 | B1801 | B4001 | Cw0304 | Cw0701 |
| SK-MEL-25 | A0101 | A0301 | B0702 | B1801 | Cw0701 | Cw0702 |
| SK-MEL-44 | A0201 |  | B1803 | B4001 | Cw03 | Cw07 |

HLA-A B18, B40 and B7 are recognized by the Bw6 mAb, but not B51 or B52.
